# Identifying Fraudulent Responses in a Study Exploring Delivery Options for Pregnancies Impacted by Gestational Diabetes: Lessons Learned From a Web-Based Survey

**DOI:** 10.2196/58450

**Published:** 2025-01-20

**Authors:** Emma Ruby, Serine Ramlawi, Alexa Clare Bowie, Stephanie Boyd, Alysha Dingwall-Harvey, Ruth Rennicks White, Darine El-Chaâr, Mark Walker

**Affiliations:** 1 Faculty of Medicine University of Ottawa Ottawa, ON Canada; 2 Clinical Epidemiology Program Ottawa Hospital Research Institute Ottawa, ON Canada; 3 Department of Obstetrics, Gynecology, and Newborn Care The Ottawa Hospital Ottawa, ON Canada; 4 Department of Obstetrics and Gynecology University of Ottawa Ottawa, ON Canada; 5 School of Epidemiology and Public Health University of Ottawa Ottawa, ON Canada; 6 International and Global Health Office University of Ottawa Ottawa, ON Canada

**Keywords:** research fraud, anonymous online research, data integrity, fraudulent responses, web-based survey, internet research, perinatal health, social media, patient participation, provider participation, fraudulent, fraud, pregnancy, gestational diabetes, diabetes, data analysis, survey, diabetes mellitus, patient, evidence-based

## Abstract

Current literature is unclear on the safety and optimal timing of delivery for pregnant individuals with gestational diabetes mellitus, which inspired our study team to conduct a web-based survey study exploring patient and provider opinions on delivery options. However, an incident of fraudulent activity with survey responses prompted a shift in the focus of the research project. Unfortunately, despite the significant rise of web-based surveys used in medical research, there remains very limited evidence on the implications of and optimal methods to handle fraudulent web-based survey responses. Therefore, the objective of this viewpoint paper was to highlight our approach to identifying fraudulent responses in a web-based survey study, in the context of clinical perinatal research exploring patient and provider opinions on delivery options for pregnancies with gestational diabetes mellitus. Initially, we conducted cross-sectional web-based surveys across Canada with pregnant patients and perinatal health care providers. Surveys were available through Research Electronic Data Capture, and recruitment took place between March and October 2023. A change to recruitment introduced a US $5 gift card incentive to increase survey engagement. In mid-October 2023, an incident of fraudulent activity was reported, after which the surveys were deactivated. Systematic guidelines were developed by the study team in consultation with information technology services and the research ethics board to filter fraudulent from true responses. Between October 14 and 16, 2023, an influx of almost 2500 responses (393 patients and 2047 providers) was recorded in our web-based survey. Systematic filtering flagged numerous fraudulent responses. We identified fraudulent responses based on criteria including, but not limited to, identical timestamps and responses, responses with slight variations in wording and similar timestamps, and fraudulent email addresses. Therefore, the incident described in this viewpoint paper highlights the importance of preserving research integrity by using methodologically sound practices to extract true data for research findings. These fraudulent events continue to threaten the credibility of research findings and future evidence-based practices.

## Introduction

Gestational diabetes mellitus (GDM) is one of the most common complications associated with pregnancy. In Canada, GDM is on the rise, with a near doubling of GDM diagnoses from 4% in 2004 to 7% in 2014 [1]. Globally, the rate of GDM is estimated to be 14.7%, according to the International Association of Diabetes and Pregnancy Study Groups [2].

Current literature is unclear on the safety and optimal timing of delivery for pregnant individuals with GDM. Canadian guidelines for delivery of GDM pregnancies differ from that of other countries, and discourse remains, even amongst health care providers [3-6]. Furthermore, literature exploring the perspectives of pregnant individuals and care providers on induction of labor (IOL) at 38 weeks compared with expectant management is limited.

Therefore, our study team initially sought to conduct a survey study exploring the topic of IOL and delivery options for pregnant individuals diagnosed with GDM. However, a significant and rapid influx of responses to the survey in mid-October 2023 prompted further investigation for fraudulent responses, shifting the focus of the study team’s objectives with this research project.

Therefore, the goal of this viewpoint paper is to highlight our experience and approach to identifying fraudulent responses in a web-based survey study in the context of perinatal clinical research regularly conducted by the study team.

## Challenges of Preserving Data Integrity in Studies With Vulnerable Populations

Particularly when conducting research with vulnerable populations such as pregnant individuals, there is a tension between maintaining participant anonymity while using techniques to prevent data fraud and protect study integrity [7]. Pregnant individuals have traditionally been excluded from research trials due to ethical concerns and misinformed ideas about clinical research [8]. Safety measures have always been at the forefront of pregnancy clinical research due to the medical complexities that pregnant individuals possess, and the additional consideration for fetal and newborn wellbeing. Therefore, providing a protected space where pregnant individuals can participate in research aimed to improve the health outcomes of themselves and their infants is integral for equitable and evidence-based practices [8]. However, breaches in data security and incident of fraudulent activity threaten this safe space.

Despite the significant rise of web-based surveys used in medical research, there remains very limited evidence on the implications of, and optimal methods to handle, fraudulent survey responses [9]. Unfortunately, there are no optimal methods or guidelines to address fraudulent incidents in research ethics or protocols [10-13]. Oftentimes, when these incidents take place, research teams must resort to less robust measures to address areas of fraud, such as filtering based on selection criteria, feasible participant timelines, duplication, repetitive responses, etc [10-13]. Therefore, challenges remain to systematically distinguish fraudulent from true responses to ensure research integrity and credibility of findings.

There is limited discourse on the incidence rates and impact of fraudulent responses to web-based surveys in the literature, making it difficult for researchers to raise awareness of these threats to data integrity. Reported incidents of data fraud in web-based studies are limited but suggest the need for rigorous protocols to distinguish fraudulent from true data to protect research integrity [7,9,11,12,14]. Less sophisticated methods used to differentiate fraud from true data have included tracking timestamps and time to completion of survey responses, using the Completely Automated Public Turing Test to Tell Computers and Humans Apart (CAPTCHA), and including several required open-ended questions to identify unusual responses [13,15,16]. More sophisticated methods have included logic checks to assess survey respondents’ attentiveness, using skip logic, or using redundant questions to ensure consistency of responses to demographic questions [13,15,16].

## Back to the Beginning: Methods for a Pregnancy Survey Study

Our team, composed of perinatal clinicians, researchers, and practicing obstetricians and gynecologists, originally sought to conduct a survey study exploring the perspectives of pregnant individuals and health care providers on their willingness to participate in research studies comparing elective IOL at 38 weeks of gestation versus expectant management for individuals diagnosed with GDM. However, we shifted our focus to explore the challenges of preserving data integrity in web-based research studies upon the discovery of a fraudulent incident with our survey responses.

## Ethical Considerations

Ethics approval was obtained from the Ottawa Health Science Network Research Ethics Board (protocol ID: 20230075-01H). We updated recruitment to allow for compensation in the means of a CAD $5 (US $3.50) gift card to a local coffee shop upon completion of the survey for eligible participants. This required that participants provide their email address in a separate survey to which a link was made available upon completion of the main survey. We advertised this monetary incentive on all recruitment materials and in the participant consent form upon accessing the web-based survey. Any information provided by participants was kept strictly confidential and only available to the research team.

## Participant Sample

Our initial survey study included 2 study groups: a patient population and a provider population. Patients were eligible to participate if they were currently pregnant with a diagnosis of GDM at the time of survey completion or up to 12 months postpartum with a diagnosis of GDM in their most recent pregnancy. Providers were eligible if they routinely provided care to the pregnant population and held a professional status that allowed them to counsel pregnant people on delivery recommendations. We sought to recruit a target sample size of 100 participants in each group (100 patients and 100 providers), totaling 200 participants. This sample size was selected to optimize study feasibility while sufficiently capturing the perspectives of pregnant people with GDM and health care providers.

## Methods of Recruitment

We used convenience and snowball sampling to recruit participants. We conducted recruitment using various strategies, including posters in hospitals, social media posts on Twitter and Facebook, active recruitment at in-person clinics, review of electronic medical records, and the leverage of existing professional colleagues of the study team.

## Survey Elements

We developed web-based surveys using Research Electronic Data Capture (REDCap) software and they took approximately 10 minutes to complete [17,18]. REDCap is a secure, web-based software platform designed to support data capture for research studies [17,18]. The surveys were cross-sectional in nature and were available as 2 different URL links (1 for each study group). The surveys were publicly available, thus anyone with access to the URL links had access to the surveys.

## Survey Measures

Data was measured using a combination of Likert scale responses, free text response options, and multiple-choice questions. Data pertaining to demographic characteristics and likelihood to participate in or support a future randomized trial comparing induction of labor and expectant management for pregnancies impacted by GDM was obtained for both patient and provider groups.

## Security Measures

In our survey study, to dissuade automated fraudulent responses, we included the CAPTCHA test, made available through the REDCap system. The URL links were made secure by generating them in REDCap, a data capture software that supports data security for research studies. In addition, the surveys were created in such a way that individuals deemed ineligible would not be allowed to advance to the next questions. This was accomplished using forced fields to ensure only eligible participants continued to survey questions.

## The Incident and Aftermath

The GDM-related surveys were initially launched on March 9, 2023, and remained active until mid-October, 2023. Upon approval of an amendment on October 4, 2023, surveys were updated on October 11, 2023, to include a monetary incentive.

Part of our data management plan included a team member monitoring the survey responses on a regular weekly basis. Therefore, we were able to identify the onset of the fraudulent incident within a short timeframe. Once it was determined that the rapid influx of responses between October 14 and 16, 2023, was in fact a fraudulent incident, the research team swiftly deactivated the survey that same day and removed posters from clinical settings as well as social media posts. Our team reported the incident to the OHSN-REB for guidance on the next steps to address the fraudulent survey responses. The information technology teams were also alerted to help to address fraudulent responses.

**Figure 1:**
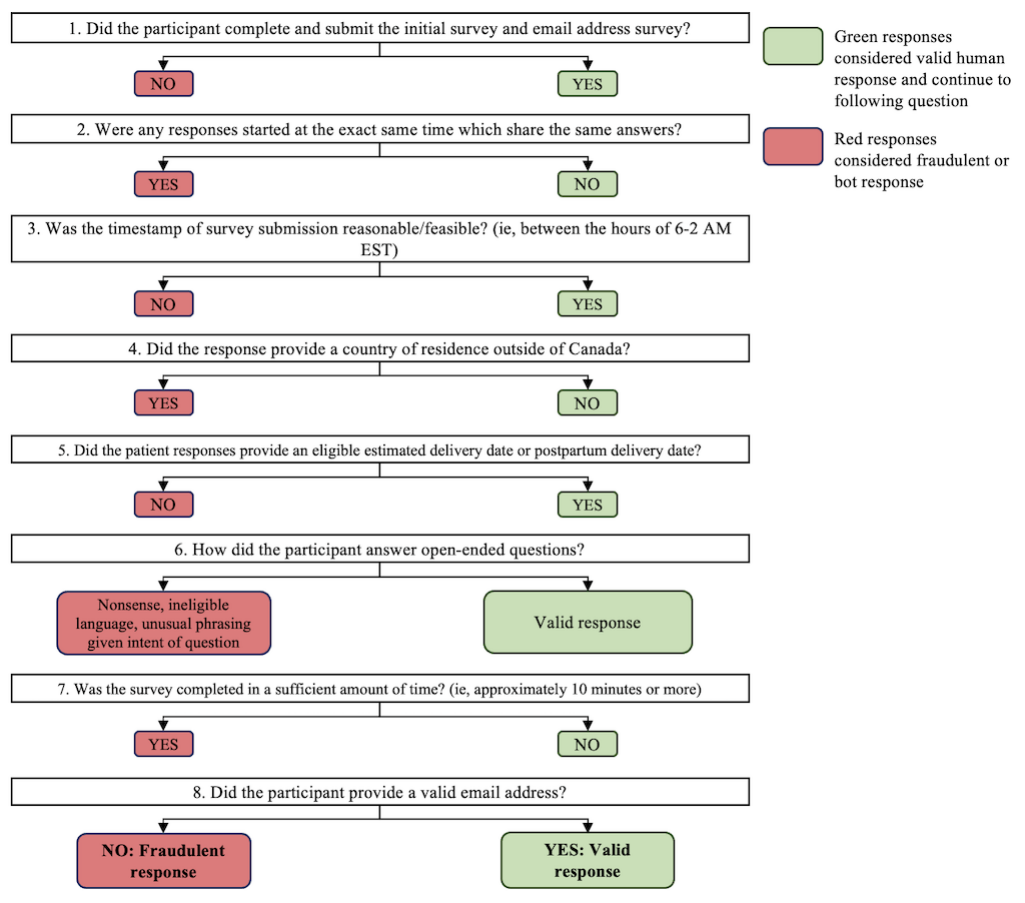
Approach to fraudulent response filtering.

We created a systematic guideline to filter fraudulent responses from true responses, by exporting the data from REDCap into Microsoft Excel and using criteria to indicate reasons for flagging responses as fraudulent (Figure 1). The responses were tallied according to their criteria and are summarized in the results.

## Our Findings

**Figure 2:**
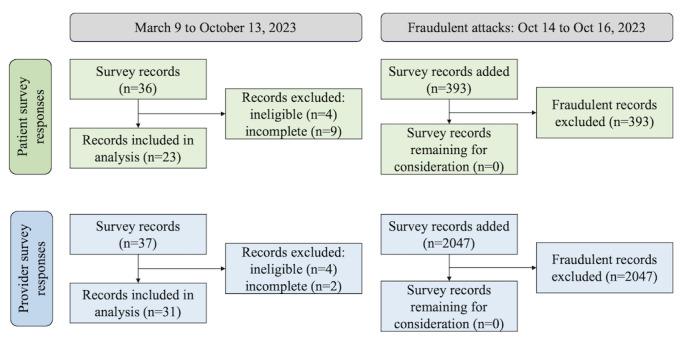
Timeline for survey response influx and screening. See Multimedia Appendix 1 for reasons why patient survey records were flagged as fraudulent. See Multimedia Appendix 2 for reasons why provider survey records were flagged as fraudulent.

From March 9 to October 13, 2023, we had a total of 36 patient responses and 37 provider responses to the survey. Upon preliminary screening, 23 patient responses and 31 provider responses were deemed eligible and included in our analysis. Between October 14 and October 16, 2023, there were an additional 393 patient survey records and 2047 provider survey records submitted (Figure 2). Given the rapid influx of responses in such a short timeframe and the nature of our recruitment before this event, our team determined this to be an incident of fraudulent activity and used a systematic guide to identify fraudulent responses. Possible reasons for the drastic difference in the ratio of patient to provider fraudulent responses included: (1) the provider survey was shorter than the patient survey, (2) the provider survey asked fewer demographic questions, and (3) the provider survey required that fewer eligibility questions be completed to advance to the next survey questions.

In consultation with the research ethics board and IT services, our team constructed a system to flag responses according to their fraudulent activity criteria (Figure 1). Criteria for filtering according to fraudulent versus true responses were applied. Following advice from the research ethics board, the surveys were inactivated and the study was closed to prevent further potential compromise to our results. Due to the limited discourse that exists on optimal methods for handling fraud in survey research, there was no obvious software that our team determined was best suited for addressing the incident. Therefore, we used Microsoft Excel files and applied a multireviewer, multidisciplinary, and multistep approach to filter fraudulent responses.

We reported reasons for why patient responses were flagged as fraudulent and tallied our findings using frequencies and percentage descriptive statistics (Multimedia Appendix 1). Reasons included an ineligible country provided or country not provided, ineligible estimated delivery date or postpartum delivery date (eg, date listed as “2004-02-01” or “1988-10-25,” which did not fall within the eligible 12 months postpartum as outlined in the eligibility criteria), same timestamp with exact same responses, same timestamp and similar responses with only slight variations in wording, responses not aligned with the intent of questions, fraudulent email address provided, and suspicious timestamp (eg, a multitude of responses being submitted between the hours of 2-6 AM Eastern Standard Time).

Reasons for why provider responses were flagged as fraudulent were also tallied and reported (Multimedia Appendix 2). Reasons included same timestamp with exact same responses, same timestamp and similar responses with only slight variations in wording, similar timestamp and similar responses, ineligible language, fraudulent email address provided, suspicious timestamp, and incomplete responses.

Criteria used to filter out fraudulent responses was defined in consultation with study team members. For example, the criteria “same timestamp and responses” was defined as any response that was written the exact same way with the same wording and submitted at the same time as any other response. Fraudulent email addresses were determined using the built-in Excel data validation function and email verification software to identify invalid mailboxes, invalid domains, spam traps, and syntax errors.

Given the significant number of fraudulent responses and the limited number of responses deemed to be “true,” the research team decided against conducting a between-group analysis of the responses included compared with those excluded.

## Fraudulent Online Bots: A Black Box in Medical Research

This paper contributes to an unfortunately limited body of evidence supporting the need for increased data security measures when conducting medical research in the technological era. Despite the significant rise of web-based surveys used in medical research, there remains very limited evidence on the implications of, and optimal methods to handle, fraudulent survey responses [9]. However disappointing, adverse fraudulent events will likely continue to occur. These events therefore obscure the true results of medical research and pose a threat to the integrity of future evidence-based practices.

Unfortunately, there are no optimal methods or guidelines to address fraudulent incidents in research ethics or protocols [10-13]. Oftentimes, when these incidents take place, research teams must resort to less robust measures to address areas of fraud, such as filtering based on selection criteria, feasible participant timelines, duplication, repetitive responses, etc [10-13]. Therefore, challenges remain to systematically distinguish fraudulent from true responses to ensure research integrity and credibility of findings.

Previous literature highlights the importance of security measures to ensure the integrity of web-based survey responses. Such security measures have included using CAPTCHA to distinguish humans from bot responses, using software that collects IP addresses of its survey user, requiring the completion of an open text response to assess individuality, or requiring email verification for the completion and submission of the survey [9,11,14,19]. Furthermore, measures to avoid the attention of fraudulent respondents may include keeping recruitment periods short and avoiding advertisement of the survey in public spaces such as on social media or on posters in the hospitals [19]. Incidents such as these indicate the need for study protocols to outline contingency plans in the case of cyber-attacks that may compromise the credibility of their findings [11,19]. Manual validation, in addition to the automatic validation techniques used to filter duplicate survey responses, should be implemented and strengthened in future web-based research studies [11,19].

We recognize that many factors likely contributed to the influx of fraudulent activity. For example, the amendment to include a monetary incentive was activated at the end of a work week, providing more weekend time for individuals to submit responses. In addition, the survey was available as a single public link rather than a disposable link, providing a convenient means to share the surveys amongst other potential users. Another limitation to our study, and what was a potential factor for the influx of fraudulent responses, was the limited eligibility criteria, since patients were eligible if they were currently pregnant, or had been pregnant in the past 12 months, and diagnosed with GDM. Due to this restriction in selection criteria and our desired sample size of 200 participants, the recruitment period was prolonged to obtain an adequate sample size, increasing the risk for fraudulent responses to be submitted to the survey. However, despite evidence to suggest that recruitment of pregnant people in research studies is most effective through face-to-face means, our team determined that using multiple recruitment strategies would limit selection bias and increase the likelihood of reaching our desired sample size [13,20-22]. Finally, REDCap software, although useful for many research studies, does not collect the IP addresses of participants accessing surveys generated through its system. This posed a limitation to our team’s ability to use robust methods to decipher fraudulent from true responses.

This viewpoint paper is unique in that it brings to light a rising concern for data security and research integrity in an era of mounting technological involvement in medical research. It highlights the real and raw experience of a group of medical researchers who pride themselves on conducting methodologically sound and scientifically robust perinatal health research. Therefore, this paper provides an example of the vulnerability of web-based medical research, and the extent of precautions that are necessary to prevent such incidents from occurring.

## Conclusions

Overall, this fraudulent incident highlights the importance of preserving research integrity by using methodologically sound practices to extract true data for research findings. Despite numerous measures used to avoid fraudulent data in medical research, findings may still be compromised, particularly in web-based studies. We hope this case example provides future researchers with a cautionary tale to consider when conducting their own survey studies. Researchers must be aware of and actively work to prevent potential threats to the credibility of their research findings and future evidence-based practices.

## Appendix

Multimedia Appendix 1 Frequencies and percentages of criteria for flagged fraudulent responses for gestational diabetes mellitus patient survey.

Multimedia Appendix 2 Frequencies and percentages of criteria for flagged fraudulent responses for gestational diabetes mellitus provider survey.

## Abbreviations

CAPTCHA: Completely Automated Public Turing Test to Tell Computers and Humans Apart
GDM: gestational diabetes mellitus
IOL: induction of labor
REDCap: Research Electronic Data Capture
