# Supplementary material for: Identifying Fraudulent Responses in a Study Exploring Delivery Options for Pregnancies Impacted by Gestational Diabetes: Lessons Learned From a Web-Based Survey
Source: J Med Internet Res. 2025 Jan 20;27:e58450. doi: 10.2196/58450 (PMC11791437; doi:10.2196/58450)
Supplement: Multimedia Appendix 1 [file jmir_v27i1e58450_app1.docx]

Multimedia Appendix 1. Frequencies and percentages of criteria for flagged fraudulent responses for GDM ***patient*** survey.

| Reason flagged as fraud | Number of responses (n=393) | Percentage (%) |
| --- | --- | --- |
| Ineligible country/country response not provided | 9 | 2.29 |
| Ineligible EDD or postpartum DD | 12 | 3.05 |
| Same timestamp and same responses | 180 | 45.8 |
| Same timestamp and similar responses with slight variations in wording | 126 | 32.1 |
| Responses not aligned with intent of questions | 6 | 1.53 |
| Fraudulent email address provided | 46 | 11.7 |
| Suspicious timestamp | 36 | 9.16 |
| Insufficient time to completion of survey (<10 mins) | 24 | 6.10 |
| Vague responses with odd/unusual phrasing | 4 | 1.02 |
