# Supplementary material for: Identifying Fraudulent Responses in a Study Exploring Delivery Options for Pregnancies Impacted by Gestational Diabetes: Lessons Learned From a Web-Based Survey
Source: J Med Internet Res. 2025 Jan 20;27:e58450. doi: 10.2196/58450 (PMC11791437; doi:10.2196/58450)
Supplement: Multimedia Appendix 2 [file jmir_v27i1e58450_app2.docx]

Multimedia Appendix 2. Frequencies and percentages of criteria for flagged fraudulent responses for GDM ***provider*** survey.

| Reason flagged as fraud | Number of responses (n=2,047) | Percentage (%) |
| --- | --- | --- |
| Same timestamp and exact same responses | 173 | 8.45 |
| Same timestamp and similar responses with slight variations in wording | 25 | 1.22 |
| Similar timestamp and similar responses to subsequent records | 183 | 8.94 |
| Ineligible language/unable to understand language | 23 | 1.12 |
| Fraudulent email address provided | 889 | 43.4 |
| Suspicious timestamp (to which number of responses would be unlikely) | 702 | 34.3 |
| Incomplete responses | 36 | 1.76 |
| Insufficient time to completion of survey (<10 mins) | 12 | 0.59 |
| Suspicious email address | 4 | 0.19 |
